# Could Insect Frass Be Used as a New Organic Fertilizer in Agriculture? Nutritional Composition, Nature of Organic Matter, Ecotoxicity, and Phytotoxicity of Insect Excrement Compared to *Eisenia fetida* Vermicompost

**DOI:** 10.3390/insects17020142

**Published:** 2026-01-27

**Authors:** Patricia Castillo, José Antonio Sáez-Tovar, Francisco Javier Andreu-Rodríguez, Héctor Estrada-Medina, Frutos Carlos Marhuenda-Egea, María Ángeles Bustamante, Anabel Martínez-Sánchez, Encarnación Martínez-Sabater, Luciano Orden, Pablo Barranco, María José López, Raúl Moral

**Affiliations:** 1 Unit of Microbiology, Department of Biology and Geology, CITE II-B, Agrifood Campus of International Excellence CeiA3, CIAIMBITAL, University of Almeria, 04120 Almeria, Spain; pcm412@ual.es (P.C.); mllopez@ual.es (M.J.L.); 2 Institute for Agri-Food and Agro-Environmental Research and Innovation (CIAGRO-UMH), GIAAMA Research Group, University Miguel Hernández, Carretera de Beniel Km 3.2, 03312 Orihuela, Spain; jose.saezt@umh.es (J.A.S.-T.); jandreu@umh.es (F.J.A.-R.); marian.bustamante@umh.es (M.Á.B.); e.martinezs@umh.es (E.M.-S.); pbvega@ual.es (P.B.); raul.moral@umh.es (R.M.); 3 Campus de Ciencias Biológicas y Agropecuarias, Universidad Autónoma de Yucatán, Mérida 97150, Mexico; hector.estrada@correo.uady.mx; 4 Department of Agrochemistry and Biochemistry, Multidisciplinary for Environmental Studies Ramón Margalef, Carretera San Vicent del Raspeig, 03690 Alicante, Spain; frutos@ua.es; 5 Department of Environmental Sciences and Natural Resources, Carretera San Vicent del Raspeig, 03690 Alicante, Spain; anabel.martinez@ua.es

**Keywords:** *Acheta domesticus*, ecotoxicity, *Galleria mellonella*, germination index, insect manure, t, FTIR, TGA/DSC, *Hermetia illucens*, solid-state ^13^C NMR, *Tenebrio molitor*, vermicompost

## Abstract

The expanding insect farming industry generates up to 67,000 tons of frass per year. Its potential use as fertilizer is promising, but has not yet been widely studied. This study aimed to characterize the chemical composition, organic matter structure, ecotoxicity, and phytotoxicity of frass from four insect species in order to evaluate its potential as a fertilizer. We compared four types of insect frass (IF) (*Tenebrio molitor*, *Galleria mellonella*, *Hermetia illucens*, and *Acheta domesticus*) to *Eisenia fetida* vermicompost (EFV). We used physicochemical analyses (pH, electrical conductivity (EC), macro-micronutrients and dissolved organic carbon (DOC), spectroscopy (solid-state ^13^C nuclear magnetic resonance (NMR), and Fourier-transform infrared spectroscopy (FTIR)) and thermogravimetry/differential scanning calorimetry (TGA/DSC: R_1_, R_2_, Tmax), together with phytotoxicity (germination index, %GI) and ecotoxicity (toxicity units, TU) bioassays. Composition was species-dependent: *A. domesticus* showed the highest levels of nitrogen (N), phosphorus (P), and potassium (K); the concentration of DOC was higher in insect frass (IF) than in EFV, with the highest concentration found in IF of *T. molitor*. ^13^C NMR/FTIR profiles distinguished between frass (carbohydrates/proteins and chitin signals) and EFV (humified, oxidized matrix). Thermal stability followed: *G. mellonella* (R_1_ ≈ 0.88) ≥ *A. domesticus* (0.79) > *H. illucens* (0.73) > EFV (0.67) > *T. molitor* (0.50). In bioassays, *T. molitor* and *A. domesticus* exhibited phytotoxicity (%GI < 30), whereas *G. mellonella* and *H. illucens* did not. EFV exhibited the highest %GI. Dilution increased %GI in all materials, especially in *T. molitor* and *A. domesticus,* and reduced acute risk (TU). Frass is not a uniform input: its agronomic performance emerges from the interaction between EC (ionic stress), the availability of labile C (DOC, C/N and low-temperature exotherms), and structural stability (R_1_/R_2_ and aromaticity). In terms of formulation, IF can provide nutrients that mineralize rapidly, whereas EFV contributes stability. Controlling the inclusion and dilution of materials (e.g., limiting the amount of *T. molitor* in blends) and considering the mixing matrix helps to manage phytotoxicity and ecotoxicity, and realize the fertilizer value of the product.

## Simple Summary

Large amounts of “frass” (insect excreta mixed with shed skins and feed residues) are generated by insect farming and this might be used as an amendment. We compared frass from four insects (mealworms, waxworms, black soldier flies, and crickets) and compared them with worm compost (vermicompost). Using a combination of chemical and spectroscopic analyses together with thermal tests and seed-based bioassays, we identified clear, species-specific differences that are important for field use. Frass from mealworms and crickets was so concentrated that it could harm plants unless it was diluted or blended, whereas frass from the other species was much gentler. Our research shows that frass acts as a rapid-action fertilizer, providing a quick nutrient boost, whereas vermicompost is better for building long-term soil health. The most effective approach is to combine the two; a small amount of frass provides plants with an immediate nutrient boost, while vermicompost creates healthy soil and protects the plants, effectively transforming insect waste into a safe and valuable organic fertilizer.

## 1. Introduction

With around 2000 edible species identified and regularly consumed worldwide [1], insect production is a rapidly expanding industry. An estimated production of 67,000 tons of insects is raised on farms each year for food and animal feed. The most farmed species are the black soldier fly (*Hermetia illucens*), the yellow mealworm (*Tenebrio molitor*), and the house cricket (*Acheta domesticus*) [2]. Other species, such as the greater wax moth (*Galleria mellonella*), are of interest as model organisms for studying microbial infections, host–pathogen interactions and virulence mechanisms, as well as for testing the toxicity of antimicrobial drugs [3,4].

The black soldier fly and the yellow mealworm are valued for their ability to efficiently convert organic waste into high-quality biomass, which is used as a protein source for animals, providing solutions to the growing demand for food [5,6,7,8]. *H. illucens*, native to America, has spread worldwide, while *T. molitor*, believed to have originated in the Mediterranean region, is now found everywhere [9]. Although historically considered pests of stored products, their bioconversion capacity has transformed them into a key asset for the circular economy. *A. domesticus*, which is also globally distributed, is a key species in food production due to its high nutritional value and its use in the manufacturing edible products. Although not widely used for food, *G. mellonella* is a model organism of great importance in biomedical and microbiological research. Its status as a bee hive pest has prompted research into its biology and control, and its characteristics make it invaluable for researching microbial infections and testing the toxicity of new drugs thanks to its innate immune system, which is similar to that of mammals [10,11], and *T. molitor* are common pests of stored products, causing substantial losses in the food and grain storage industries [12]. This duality underscores the need for a thorough understanding of their biology in order to harness their beneficial applications while developing effective strategies to mitigate their harmful impact.

IFs are produced in large quantities due to the growing insect farming industry, particularly from species such as mealworms and black soldier flies. This makes them a promising source of fertilizers or even more [13,14]. The composition of IF varies among insects; for instance, black soldier fly frass is particularly rich in nitrogen and potassium, while cricket frass tends to have a higher phosphorus content [15,16,17]. Producing IF also offers an opportunity to transform organic waste into a valuable source of protein and fiber for meat and fish production [18]. Production of IF can be up to 40 times greater than insect biomass [19]. In a pilot-scale production process involving black soldier fly larvae and 1400 kg of food waste, approximately 239 kg of insect biomass and 230 kg of frass were produced [20]. The advantages of using IF as fertilizers, when used properly, include higher levels of readily available macronutrients, prevention of phosphorous losses, the presence of beneficial microorganisms, promotion of plant growth, enhancement of photosynthetic efficiency, biotic and abiotic stresses, as well as ease of combination with slow-release agents and biostimulants [21,22]. However, some risks associated with the chemical and microbiological composition of IF have been documented, such as heavy metals, the presence of pathogens like *Salmonella* and *Listeria*, and mycotoxin content [23,24,25].

The composition of IF includes insect excrement, exuviae (shed skins), and undigested feed residues. The exact composition is influenced by the species of insect species and the substrate on which they feed [26]. At a molecular level, IF contains proteins, lipids, and carbohydrates [27]. It also contains fatty acids, amides, and a variety of bioactive molecules, as well as hydrocarbons with both short and long carbon chains [28]. Chitin, which originates from insect exoskeletons and shed skins, is a key organic compound in IF [29]. It has been shown to be a nitrogen source for soil fungi, as well as having nematicidal, antimicrobial, antipathogenic, and antiviral effects [30,31,32,33]. While the physical parameters and nutrient composition of IF are well understood [14,34], the structure and biodegradability of its organic compounds remain unstudied.

To address this gap, we used a combination of spectroscopic and thermal approaches to characterize the composition and stabilization of organic-matter in insect frass, benchmarking it against *E. fetida* vermicompost. ATR-FTIR spectroscopy provides rapid functional-group fingerprints (e.g., polysaccharide C–O features, amide bands related to proteins/chitin, carbonyl/carbonate signals), enabling the qualitative comparison of biochemical signatures and the degree of transformation among substrates [35,36,37]. In parallel, solid-state ^13^C CP-MAS NMR resolves carbon functional domains (e.g., alkyl, O-alkyl, aromatic and carbonyl), enabling semi-quantitative descriptors of biochemical composition (e.g., carbohydrate, protein, and lignin-like contributions) and structural features linked to the decomposition stage through established integration and mixing frameworks [38]. Finally, thermogravimetric analysis (TG/DTG) and differential scanning calorimetry (DSC) under oxidative conditions provide an independent view of organic-matter stability, tracking mass loss and exothermic reactions across temperature windows associated with labile versus more recalcitrant fractions. The derived indices (e.g., R_1_ from TG mass-loss ratios, and R_2_/Tmax descriptors from DSC) can be used as proxies for organic-matter stabilization and thermal recalcitrance. They are particularly informative when interpreted alongside spectroscopic fingerprints [39,40,41]. By integrating these instrumental descriptors with physicochemical traits and bioassays (phytotoxicity and ecotoxicity), this study links species-specific organic-matter composition and stability to biological outcomes. It also proposes a more diagnostic basis for defining safe, formulation-oriented management guidelines for the use of insect frass use in agriculture.

In moderate doses, IF enhances soil fertility, invertebrate and microbial activity and plant growth [29,42,43]. However, the phyto- and ecotoxicological effects of IF must be considered when using it as an organic fertilizer or as an ingredient in a complex fertilizer. It is therefore crucial to consider the potential negative effects of the excessive or unregulated application of these IF, including the inhibition of germination, mycorrhization, and plant growth due to ammonia toxicity, salinity, or high nutrient concentrations [44,45], as well as the risk of contamination by mycotoxins, dioxins, heavy metals, pesticide residues, veterinary drugs, and human foodborne pathogens [23,46]. This study compares frass from *A. domesticus*, *H. illucens*, *G. mellonella*, and *T. molitor* with *E. fetida* vermicompost (EFV), with the aim of: (i) quantifying nutrient and salinity traits, (ii) characterizing organic-matter composition and thermal stability (^13^C NMR, FTIR and TGA/DSC), and (iii) assessing phytotoxicity and ecotoxicity. The aim is to define species-specific management guidelines for the safe agricultural use of these materials.

## 2. Materials and Methods

### 2.1. Sampling of Insects of Study

**Figure 1:**
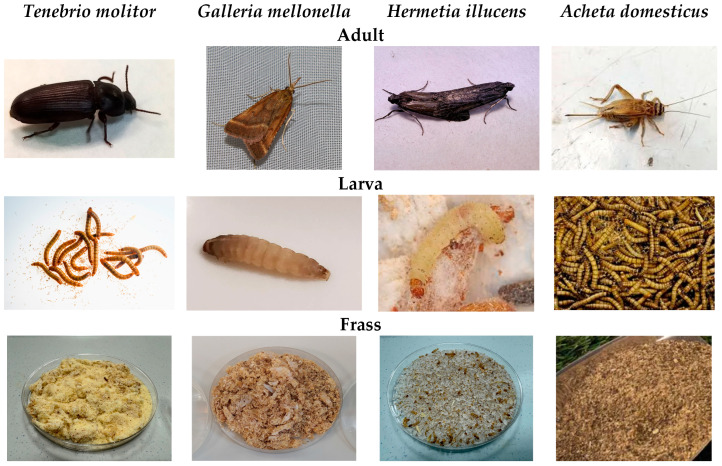
Detailed images of studied insect species (adults and larvae) and their associated frass.

Samples were collected from commercial and research sites the UAL Entomologic Center (Almería, Spain), the ENTOGREEN^®^ (Santarém, Portugal) and NUTRINSECT^®^ (Montecassiano, Italy). Additionally, ten EFV samples of different EFV from the UMH-EPSO (Oirhuela, Spain) were collected as reference material for comparison with IF (Figure 1). All collected samples were dried at 60 °C, milled, and sieved to 0.5 mm prior to analysis.

### 2.2. Assessment of the Nature and Composition of Ifs and EFVs

IFs and EFVs were analyzed according to the methods described by Martín et al. [37]: electrical conductivity (EC) and pH were analyzed in a 1:10 (*w*/*v*) water extract. Total organic matter (TOM) was determined by loss-on-ignition at 430 °C for 24 h, and total nitrogen (TN) and total organic carbon (TOC) were measured by ignition at 1020 °C using automatic elemental microanalyzers (EuroVector elemental Analyzer, Milano, Italy). The humic (C_HA_) and fulvic (C_FA_) content was measured according to the method described in [47]: after the 1:20 alkaline (NaOH) extraction using an automatic analyzer for liquid samples (TOC-VCSN Analyzer, Shimadzu, Kyoto, Japan) was used. After microwave acid digestion, total elemental content of P, K, Ca, Mg, Na, and heavy metals were analyzed by ICP-OES.

### 2.3. Biological Parameters

The germination index (GI) of the obtained compost was measured by assessing the germination and radicle length of garden cress (*Lepidium sativum* L.) was assessed according to the method of [48]. The Aboatox kit (kit 1, 243–500 BioTox; Aboatox, Masku, Finland) was used to reconstitute *Aliivibrio fischeri* following the manufacturer’s protocol. A luminometer (Luminoskan Ascent; Thermo Fisher Scientific, Waltham, MA, USA) was used to measure luminescence. The *A. fischeri* ecotoxicity assay was performed in triplicate (*n* = 3). This level of replication is commonly used for this highly standardized and low-variability bioassay and is consistent with the experimental design principles outlined in ISO 11348-3:2007 [49]. (Water quality—Determination of the inhibitory effect of water samples on the light emission of *Vibrio fischeri*). The sample concentration producing a 50% decrease in *A. fischeri* light emission (EC_50_) was calculated from the corresponding regression equations. Toxicity units (TU) were calculated using the formula TU = [1/(EC_50_)] × 100.

### 2.4. Solid-State ^13^C NMR Spectroscopy, Fourier-Transform Infrared (FTIR) Spectroscopy, Thermogravimetric Analysis (TG/DTG) and Differential Scanning Calorimetry (DSC)

Samples were analyzed by solid-state ^13^C CP-MAS NMR (cross-polarization magic angle spinning carbon-13 nuclear magnetic resonance) using a Bruker spectrometer (Bruker AVANCE DRX500, Billerica, MA, USA) operating at 125.75 MHz for ^13^C. The samples were packed in a 4 mm diameter zirconium oxide rotor with Kel-F buffers and rotated at 2000 ± 100 Hz. The optimal contact time for these samples in the CP-MAS experiments was 1.0 ms. A total of 4000 scans were acquired, with an inter-scan time of 1.5 s, and the spectra were processed with an LB (line broadening) of 20 Hz. The special distribution in the range of carbon chemical shifts was calculated by integrating the signal into seven regions: carbonyl (210–160 ppm), O-aromatic (160–140 ppm), aromatic (140–110 ppm), O2-alkyl (110–90 ppm), O-alkyl (90–65 ppm), N-alkyl/methoxyl (65–45 ppm), and alkyl (45 to −10 ppm) [38]. This only indicates the predominant type of carbon in each region.

FTIR spectra were obtained from the samples using a Bruker IFS 66 spectrometer. The resolution was set to 4 cm^−1^ and the operating range was 600 to 4000 cm^−1^. The analytical technique used was attenuated total reflection (ATR) FT-IR spectroscopy. The spectra were collected using an ATR accessory, which presses the sample onto a diamond crystal. The spectrum of each sample was obtained from an average of 20 scans, with background correction against air. Functional group assignments were made according by [50].

Thermal stability was assessed using a thermogravimetric analyzer under oxidative conditions in a Mettler Toledo (TGA/SDTA851e/LF/1600) (Greifensee, Switzerland). Approximately 10 mg of the sample was heated from room temperature to 700 °C at a rate of 10 °C/min. The TG and DTG curves were analyzed to identify the stages of mass loss, and the R_1_ index was calculated as the ratio of the mass loss in the 400–550 °C range to that in the 200–400 °C range. This is a robust proxy for assessing the degree of organic matter recalcitrance [40,41]. DSC was performed under an air atmosphere using the same heating program as TG. Exothermic peaks were used to determine the temperatures of maximum thermal decomposition (Tmax) in two ranges (200–400 °C and 400–550 °C).

### 2.5. Statistical Analysis

The ecotoxicity data, expressed as toxicity units (TUs), were analyzed using a one-way ANOVA to assess the differences between the frass. Prior to analysis the assumptions of normality and homogeneity of variances were verified. When significant differences were detected (*p* < 0.05), the least significant difference (LSD) test according to Fisher was applied for pairwise comparisons. The results are displayed as boxplots, and each frass type was evaluated using three independent replicates (*n* = 3). Statistical analyses were performed using Statgraphics Centurion version 19.2.01 (StatPoint Technologies, Inc., Warrenton, VA, USA).

One-way ANOVA was used to analyzed the physicochemical data obtained for IF and EFV, followed by the LSD test (*p* < 0.05). The Shapiro–Wilk and Levene tests were used to check for normality and homogeneity of the variances, respectively, prior to ANOVA. All statistical analyses were performed using the IBM SPSS version 30.0 software package.

## 3. Results

### 3.1. Chemical Composition of IF

Table 1 shows the elemental composition of frass from four insect species (*T. molitor*, *G. mellonella*, *H. illucens*, and *A. domesticus*). Significant differences (*p* < 0.001) were observed among the frass types for all analyzed macronutrients. The highest C concentration (41.9%) was found in *G. mellonella* frass, while the lowest was found in *A. domesticus* frass (34.8%). The nitrogen content was highest in *A. domesticus* frass (6.4%) and lowest in *H. illucens* frass (2.9%). This resulted in a C/N ratio that was lowest for *A. domesticus* frass (5.4) and highest for *H. illucens* frass (12.6). Frass derived from *A. domesticus* consistently exhibited the highest concentrations (*p* < 0.001) of phosphorus (P, 1.44%), potassium (K, 3.02%), calcium (Ca, 2.21%), magnesium (Mg, 0.66%), sodium (Na, 0.60%), and sulfur (S, 0.53%). The lowest concentrations of P (0.77%) and K (0.99%) were recorded in *G. mellonella* frass. *T. molitor* frass exhibited the lowest Ca content (0.19%) and Na content (0.03%). *G. mellonella* frass showed the lowest Mg content (0.15%), and *T. molitor* frass had the lowest S content (0.21%). The Si concentration was significantly higher (*p* < 0.001) in *G. mellonella* frass (1.32%) than in the frass of the other species. Statistically significant differences (*p* < 0.001, unless otherwise specified) were found for micronutrients and most trace elements across the different frass samples. The highest levels of Fe (871 ppm), Cu (66.2 ppm), Mn (473 ppm), Zn (587 ppm), Mo (6.37 ppm), Ni (9.4 ppm), Cr (11.0 ppm), Al (871 ppm), Cd (0.17 ppm), Pb (1.03 ppm), Co (1.03 ppm), Ti (27.1 ppm), Sr (36.9 ppm), and Rb (25.4 ppm) were found in *A. domesticus* frass. *G. mellonella* frass was the richest in B (756 ppm) and Be (0.16 ppm), but contained the lowest amounts of Fe (42 ppm), Cu (4.3 ppm), Mn (39 ppm), Zn (57 ppm), and Mo (0.27 ppm). The lowest Ni (1.2 ppm) and Cr (1.5 ppm) concentrations were found in *T. molitor* frass. *H. illucens* frass showed the lowest level of Cd (0.04 ppm). A general comparison of IF with EFV revealed higher levels of C, N, and S, but lower levels of Ca, Fe, Cu, Mn, and Zn. Other micronutrients and trace elements were present in low concentrations in both types of organic materials. The fulvic and humic carbon values determined in IF were statically higher than those in EFV (Table 2).

### 3.2. Nature of Organic Matter in IF Compared to EFV

The physicochemical profiles of IF and EFV are reported in Table 2. Statistical differences in pH were found between the various insect frass and EFV samples. Frass from *G. mellonella*, *A. domesticus*, and especially *T. molitor* had a pH value below 7, while the frass from *H. illucens* and EFV were slightly alkaline with values above 7. With the exception of *A. domesticus* frass, all the IF samples exhibited EC values similar to those of EFV (2.30–5.77 dS m^−1^). The content of dissolved organic carbon content is much higher in insect frass than in EFV. Furthermore, we observed a statically significant difference when comparing the different IF samples. The frass from *A. domesticus* and *G. mellonella* had similar DOC content, while the DOC of *H. illucens* was intermediate, and the DOC content of *T. molitor* was twice that of *A. domesticus* and *G. mellonella*.

### 3.3. Spectral and Structural Composition

**Figure 2:**
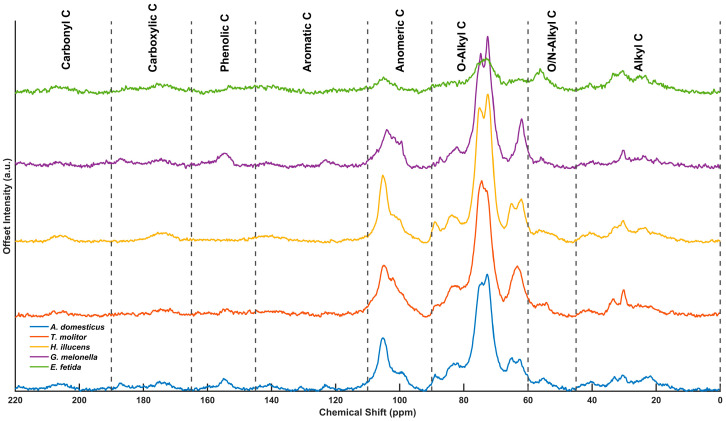
Stacked ^13^C CP-MAS NMR spectra of the IF samples analyzed in this study. Each spectrum has been vertically offset to facilitate visual comparison of the distribution of signals across chemical shift regions. The spectral regions are annotated according to the functional group assignments: Alkyl C (0–45 ppm), O/N-Alkyl C (45–60 ppm), O-Alkyl C (60–90 ppm), Anomeric C (90–110 ppm), Aromatic C (110–145 ppm), Phenolic C (145–165 ppm), Carboxylic C (165–190 ppm), and Carbonyl C (190–220 ppm). Vertical dashed lines indicate the boundaries between regions. The variation in peak intensity and distribution across the samples reflects differences in molecular composition and the extent to which the organic matter has been transformed.

The solid-state ^13^C CP-MAS NMR spectra of IF in a solid state revealed distinctive molecular profiles, enabling detailed interpretation of the chemical structures and quality of the organic matter. Integrating spectral region quantification with the biomolecular mixing model proposed by [38] enabled the relative contributions of the major biochemical classes (carbohydrates, proteins, lignin, aliphatic, and carbonyls) to be inferred, facilitating functional comparisons among these biologically derived residues (Figure 2).

Normalized integrals from the NMR spectra revealed distinct chemical differences between the samples (Supplementary Materials: Table S1). Among the insect samples, *G. mellonella* frass displayed the highest aromaticity index (0.80) among the insect samples, driven by a notable increase in the aromatic (110–145 ppm) and phenolic (145–165 ppm) signal intensities. In contrast, the EFV exhibited a more heterogeneous and balanced spectral profile. Significant signals were detected in the alkyl (0–45 ppm), aromatic (110–160 ppm), and carbonyl (160–220 ppm) regions, which are indicative of advanced microbial reworking and humification processes [51]. These compositional differences are clearly visible in the stacked NMR spectra (Figure 2).

### 3.4. Biomolecular Composition

The estimated biomolecular composition derived from the NMR spectral distributions and modelled using the approach of [38], provides insights into the biochemical nature and decomposition stage of each substrate (Table 3, Supplementary Materials: Figure S1). IF samples were characterized primarily by high carbohydrate content, ranging from 58.92% in *G. mellonella* to 66.01% in *H. illucens*. This trend reflects the chitin–protein–polysaccharide matrix that is typical of insect excreta and exuviae [38]. Protein content was also substantial, ranging from 21.73% in *T. molitor* to 29.29% in *A. domesticus*, which supports their interpretation that insect-derived residues are rich in labile nitrogenous compounds. These biochemical features align with the nutrient-rich profile often attributed to insect frass, supporting their classification as readily mineralizable soil systems inputs.

Lignin content varied among the insect frass types. *T. molitor* exhibited the highest lignin estimate (12.98%), while *H. illucens* showed the lowest (4.75%). *G. mellonella* and *A. domesticus* had intermediate values (8.93% and 7.07%, respectively), possibly reflecting differences in digestive physiology or feedstock input. Aliphatic fractions were negligible or absent in all insect samples except *H. illucens* (3.24%) and *T. molitor* (2.27%), in which minor amounts were detected. Carbonyl content was generally low, except in *G. mellonella* (5.06%), indicating limited oxidative transformation.

### 3.5. Functional Group Characterization

**Figure 3:**
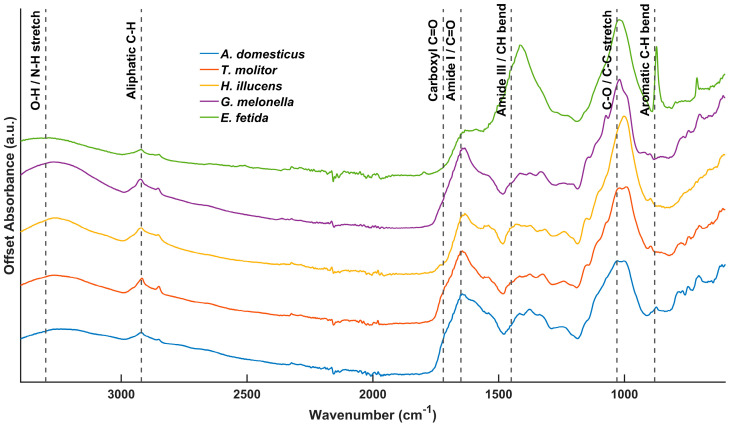
FTIR spectra of insect frass (IF) and *E. fetida* vermicompost (EFV) samples analyzed showing characteristic absorbance bands across the mid-infrared region. These spectra reveal compositional differences between substrates, which may be due to variations in organic functional groups such as proteins, lipids, and carbohydrates.

The FTIR spectra of IFs showed distinct absorption patterns across the mid-infrared region (Figure 3), reflecting the compositional diversity and biochemical specificity of these substrates. Insect frass samples exhibited strong absorption bands in the range of 998.9 to 1073.2 cm^−1^. These peaks are commonly attributed to C–O–C stretching vibrations in chitin polysaccharides and P–O–C linkages in phosphate-containing biomolecules. The presence of chitin was inferred from spectroscopic signatures [52,53], rather than being directly quantified. Their presence highlights the biochemical origin of the residues, particularly the influence of chitinous exuviae and organic material derived from the insect gut. The ^13^C NMR spectra showed clear chemical separation between the samples (Supplementary Materials: Table S1): *G. mellonella* frass had the highest aromaticity index (0.80), followed by *T. molitor* (0.56), *A. domesticus* (0.51), and *H. illucens* (0.41), while the vermicompost from *E. fetida* (EFV) had an index of 0.43. FTIR analysis revealed the presence of the polysaccharide band at ~1025.9 cm^−1^ in all samples. The frass exhibited the amide I (~1644 cm^−1^) and amide II (~1532 cm^−1^) bands, while signals showing at 871.6 and 1414.5 cm^−1^ were associated with carbonates and aliphatic deformations. Furthermore, C–H stretching vibrations at 2850.3 and 2918.7 cm^−1^ and carbonyl bands at around 1726.9 cm^−1^ were detected in all samples. Estimated biomolecular partitioning indicated a lignin content of 12.98% (*T. molitor*), 8.93% (*G. mellonella*), 7.07% (*A. domesticus*), and 4.75% (*H. illucens*) in frass, and an approximate distribution of ~30% carbohydrates, ~30% proteins, 26.43% lignin, and 13.02% aliphatic fractions in EFV.

### 3.6. Degradability Assessment

**Figure 4:**
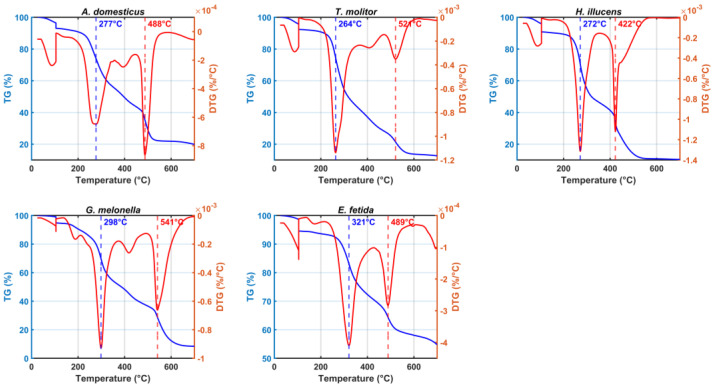
Thermogravimetric (TG) and derivative thermogravimetric (DTG) profiles for each organic matter sample. Left y-axis shows relative mass loss (%) as a function of temperature; right y-axis shows mass loss rate (%/°C). Dashed vertical lines indicate temperatures corresponding to minimum DTG signal within predefined thermal decomposition ranges of 200–400 °C and 400–550 °C. These thermal transitions reflect the sequential degradation of labile and more recalcitrant components within each sample.

The highest R_1_ value observed in the present study was in the frass from *G. mellonella* (0.88), followed by *A. domesticus* (0.79) and *H. illucens* (0.73). In contrast, *T. molitor* frass exhibited the lowest R_1_ value (0.50), indicating a predominance of labile, thermally volatile components, such as simple carbohydrates and proteins. The EFV presented an intermediate R_1_ value of 0.67, reflecting its balanced chemically composition (Figure 4, Supplementary Materials: Table S2).

### 3.7. Differential Scanning Calorimetry (DSC)

**Figure 5:**
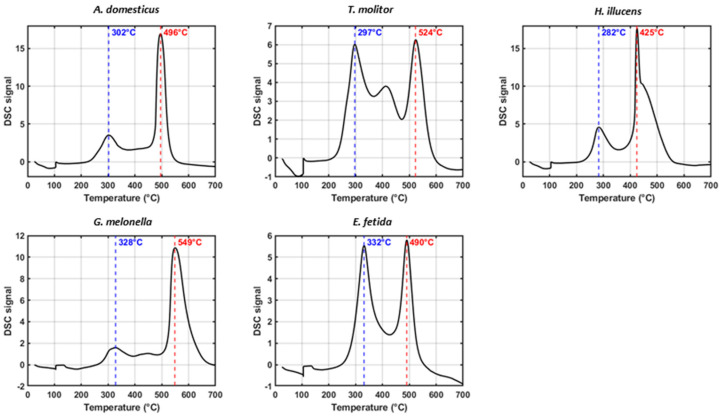
Differential scanning calorimetry (DSC) profiles of organic matter in insect frass (IF) and *E. fetida* vermicompost (EFV) samples, analyzed under controlled heating conditions. Curves show thermal transitions associated with decomposition and structural transformation of organic components. Vertical dashed lines indicate temperatures at which maximum heat flow was recorded within defined temperature ranges of 200–400 °C and 400–550 °C, respectively.

DSC complements thermogravimetric analysis by characterizing the exothermic behavior of the decomposition of organic matter under oxidative conditions. The maximum temperatures of heat release (Tmax) in two defined thermal intervals 200–400 °C (Tmax_1_) and 400–550 °C (Tmax_2_) indicate the activation energy required for the degradation of labile and recalcitrant organic fractions, respectively [29,33] (Figure 5).

The differences observed among the samples reflect variations in thermal stability and compositional features related to their biological origin and the extent of their processing. Of the insect frass samples, *G. mellonella* exhibited the greatest thermal resistance across both temperature ranges, with Tmax_1_ = 327.7 °C and Tmax_2_ = 549.0 °C. Consistent with this, *G. mellonella* also exhibited the highest R_1_ value (0.88) and the highest R_2_ value (2.55), denoting a pronounced shift in thermal decomposition towards the recalcitrant domain. The large Area_2_ (327.96) relative to Area_1_ (128.78) reinforces this interpretation, suggesting that a significant proportion of its organic matter only decomposes at elevated temperatures. In contrast, *T. molitor* displayed the lowest Tmax_1_ (297.0 °C), relatively high Tmax_2_ (523.7 °C), and the lowest R_1_ (0.50) and R_2_ (0.91). *H. illucens* frass presented the lowest Tmax_1_ (282.3 °C) and a moderate Tmax_2_ (425.0 °C), suggesting early decomposition of labile compounds with limited structural complexity. However, its relatively high Area_2_ (985.24) compared to Area_1_ (398.17) and R_2_ (2.47) implies that a substantial portion of its organic matter is associated with high-temperature degradation. *A. domesticus* exhibited intermediate thermal behavior, with Tmax_1_ = 302.3 °C and Tmax_2_ = 495.7 °C. Its Area_1_ (328.79) was lower than Area_2_ (781.22), resulting in an R_2_ of 2.38, which correlates well with its R_1_ of 0.79. For the EFV, Tmax_1_ and Tmax_2_ were 331.7 °C and 490.3 °C, respectively. The balance between Area_1_ (371.44) and Area_2_ (350.09) yielded an R_2_ value of 0.94, which is very similar to its R_1_ value of (0.67). Comparing R_1_ (from TGA) and R_2_ (from DSC) parameters across samples reveals a coherent pattern: samples with higher R_2_ values also tend to exhibit elevated R_1_ values, reflecting the dominance of late-degrading, thermally stable compounds (Supplementary Materials: Table S3).

### 3.8. Biological Parameters: Eco- and Phytotoxicity of IF

**Figure 6:**
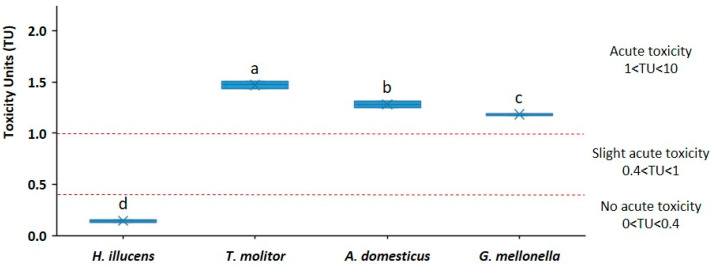
Boxplot showing ecotoxicity of insect frass (IF), expressed in toxicity units (TU = 100/EC_50_). Data are mean values (*n* = 3). Toxicity ranges are based on criteria of [53]. Different letters indicate significant differences (*p* < 0.05).

The ecotoxicity of the samples was assessed using the criteria outlined in [54]. This classification establishes toxicity categories according to Toxicity Units (TU): 0.4 < TU < 1, slight acute toxicity; 1 < TU < 10, acute toxicity; 10 < TU < 100 high acute toxicity. In this sense, the frass of *H. illucens*, 0.15 TU, presented lower ecotoxicity than that of *T. molitor* (1.47 TU), *A. domesticus* (1.28 TU), and *G. mellonella* (1.18 TU) (Figure 6).

IF and EFV were analyzed phytotoxicity using tests developed by [48]. In these tests, the germination percentage (%G) and root length percentage (%L) of the blank treatments (i.e., no frass or vermicompost) were obtained in order to calculate a combined parameter known as the germination index (%GI). The standardized threshold value for considering an organic material to be non-phytotoxic is 50% GI. Significant differences (*p* < 0.001) were observed between organism types for all three phytotoxicity indicators (Table 4). Extracts from *T. molitor* exhibited the most pronounced phytotoxicity, with extremely low values observed for germination (13.8%), radicular length percentage (8.8%), and, consequently, the germination index (1.3%). *A. domesticus* frass extract also demonstrated significant phytotoxicity, particularly inhibiting root elongation (L = 40.7%), which lead to a low germination index (28.6%). However, germination (70.3%) was less severely affected them in the case of *T. molitor*. By contrast, extracts from *G. mellonella* and *H. illucens* exhibited high germination percentages (99.1% and 85.4%, respectively) and radicular lengths (73.9% and 81.2%, respectively), resulting in germination index values (72.6% and 69.7%, respectively) that were well above the phytotoxicity threshold. Notably, the *E. fetida* vermicompost extract displayed the highest radicular length and germination index values (128% and 129% respectively).

## 4. Discussion

This study supports the view that insect frass (IF) is not a uniform input but rather a set of residues types that are structured by species and whose agronomic behavior emerges from distinct biochemical, ionic, and stability signatures. The contrasting species we studied (*A. domesticus*, *H. illucens*, *G. mellonella*, and *T. molitor)*, align with previous reports that the properties of frass depend on the biology of the insect, the feedstock, and the processing. They also differ systematically from EFV [15,55,56,57].

### 4.1. IF Agronomic Properties

Across species, IF showed nutrient densities that could exceed the effective fertilizing value (particularly for *A. domesticus* and *H. illucens*. This corroborates earlier observations of comparatively high total N and exchangeable K in some IF [58,59]. In our dataset, *A. domesticus* reached values of approximately 6.4% N and 3.0% K, values that position this frass as a potential source of rapid-nutrition component for high-demand crops, provided application rates are synchronized with crop uptake [60,61]. However, pronounced interspecific variability was observed, underscoring that “species-agnostic” dose recommendations are not defensible [25]. In practice, IF should be formulated with explicit species resolution, considering both nutrient supply and co-factors that affect bioavailability (e.g., ionic strength and labile C).

### 4.2. Environmental Issues of IF

With regard to salinity risk, previous studies have reported comparable EC ranges for IF and EFV, and have clarifies the mechanisms driving EC increases during biotransformation. Consistent with our results, ref. [62] found that the EC values of frass were similar to those of vermicompost derived from farm manure (2.30–5.77 dS m^−1^). In our dataset, only frass from *A. domesticus* would pose a salinity risk. During vermicomposting, EC typically rises as earthworms and associated microbiota decompose organic matter, releasing soluble salts [63,64]. A similar pattern has been observed during the digestion of food waste by black soldier fly larvae [65,66].

Trace metal concentrations were generally low, but not negligible, and patterns were species-specific. Although EFV often carries legacy metals from manure or mixed organics [67,68], some IF—particularly *A. domesticus* in our study—showed comparatively higher concentrations of Cd, Pb, or Ni. The normative framework for placing insect frass on the market as fertilizer is defined by the Commission Regulation (EU) 2021/1925 and Regulation (EU) 142/2011, which specifically established microbiological criteria on frass. However, requirements on chemical contaminants, such as heavy metals, are not included in these regulations. For this, the heavy metal thresholds considered are those established for organic fertilizers (Regulation (EU) 2019/1009). According to this Regulation, the maximum permissible concentrations in the final product (on a dry matter basis) are Cd: 1.5 mg kg^−1^ (for amendments) and 2 mg kg^−1^ (for fertilizers); Cu: 300 mg kg^−1^ Ni: 50 mg kg^−1^, Pb: 120 mg kg^−1^, and Zn: 800 mg kg^−1^. When compared the IF samples with these standards, it was observed that the concentrations of heavy metals were much lower than the limit values established for the safe use of organic fertilizers (Regulation (EU) 2019/1009). Such differences are attributed to the feed substrate and mineral “carry-over” during gut passage [69,70]. From a risk perspective, these interspecific differences highlight the importance of controlling the feedstock and monitoring batch-levels to ensure that metals concentrations below agronomic thresholds and to prevent long-term soil accumulation [71,72,73]. This is consistent with the premise of the circular bioeconomy that the valorization of side streams is only sustainable when quality assurance prevents the transfer of contaminants [74].

### 4.3. Organic-Matter Nature of IF

The chemical milieu of IF spanned a broad C/N and pH range exhibiting clear species structure. *A. domesticus* exhibited a very low C/N ratio of ~5.4, which is indicative of high N availability and fast turnover. In contrast, *H. illucens* and *T. molitor* presented higher C/N ratios of >11, which is consistent with slower net mineralization [75]. The pH ranged from mildly acidic (*T. molitor* ≈ 5.6) to slightly alkaline (*H. illucens* ≈ 7.5), which is within the agronomic norms for organic amendments [67,75]. Electrical conductivity (EC) was the key differentiator: *A. domesticus* displayed the highest EC among the IF and EFV, consistent with reports of elevated soluble salts in certain cricket frass depending on diet and processing [15,56]. These axes (C/N, pH, and EC) interact mechanistically with thermal stability to shape early biotic responses. Low C/N accelerates N release, but when coupled with high EC it can impose osmotic stress in salt-sensitive contexts [76]. Previous studies have described the mechanisms by which black soldier frass (BSF) digestion can neutralize humic functional groups, as well as delineating safe pH–EC ranges for agricultural use. As described by [64], *H. illucens* has the capacity to neutralize the carboxyl and phenol groups of the humic acid by secreting calcium and ammonia in the gut during digestion. Similar behavior was also observed in earthworm composting [77]. The transformation of soybean by black soldier fly larvae frass could increase the pH from 5.7 to 8.6 in the final compost [78]. The pH values (5.5–8.5) of all IF and EFV samples remained within the suitable range for earthworm and microorganism activity [79] and for agricultural and horticultural purposes [65]. EC value is one of the key parameters that could indicate the use of organic fertilizer in agriculture without environmental risk [80].

### 4.4. Structural/Molecular Nature of IF vs. EFV

Spectroscopic fingerprints (^13^C NMR and FTIR) revealed fundamental compositional differences between IF and EFV. Normalized ^13^C NMR integrals revealed distinct chemical separation among the samples (Supplementary Materials: Table S1). Notably, frass from *G. mellonella* exhibited the highest aromaticity index (0.80), driven by elevated signals in the aromatic (110–145 ppm) and phenolic (145–165 ppm) regions. This is consistent with greater contributions from recalcitrant constituents (e.g., melanin or aromatic amino acid derivatives), which are linked to species-specific biochemistry and/or digestive processing. Conversely, EFV displayed a lower aromaticity index (0.43) relative than IF, consistent with a shift from condensed aromatic domains towards more oxidized, microbially processed compounds [81]. The offset presentation highlighted the distinct functional carbon pools across the sample set. The insect-derived residues showed narrow, carbohydrate-rich profiles with dominant signals in the O-alkyl and N-alkyl domains, whereas the EFV presented a broader spectrum, reflecting the chemical diversification typical of humified organic matter. Furthermore, applying of trapezoidal integration (trapz) improved the accuracy of regional quantification by mitigating the overestimation issues associated with simple summation approaches [82,83].

These NMR trends were corroborated by FTIR features, which further supported the compositional split between IF and EFV. IF showed stronger signatures corresponding to proteins, chitin, and labile polysaccharides, whereas EFV reflected a more transformed, oxidized and microbially reworked matrix. Notably, certain insect-specific bands were absent in EFV, highlighting the distinct distribution of functional-group distribution. The ~1026 cm^−1^ band (C–O stretch in cellulose/hemicellulose) was sharper and more intense in frass, which is consistent with lower degrees of microbial alteration. The amide I (~1644 cm^−1^) and amide II (~1532 cm^−1^) were more pronounced in frass, indicating the presence of peptide bonds and residual protein. This is consistent with the NMR evidence for nitrogenous biopolymers. Although carbonyl bands near 1727 cm^−1^ appeared in all samples, implicating oxidative formation of partially degraded intermediates, the signal was stronger in *G. mellonella*, suggesting higher levels of oxidized carbon. Conversely, C–H stretching intensities at 2850/2919 cm^−1^ were lower in IF than in EFV, consistent with the comparatively reduced aliphatic fractions quantified by NMR. Across IF types, biomolecular partitions differed. *T. molitor* showed the highest lignin estimate (12.98%), *H. illucens* the lowest (4.75%) and *G. mellonella* and *A. domesticus* showed intermediate values (8.93% and 7.07% respectively). Aliphatic content was negligible across all frass samples, except for minor fractions in *H. illucens* (3.24%) and *T. molitor* (2.27%). Carbonyl content was generally low, with a higher proportion in *G. mellonella* (5.06%), indicating limited but detectable oxidative transformation.

In parallel, NMR region integrals revealed polysaccharide- and chitin-rich signatures and varying aromaticity across IF (e.g., *T. molitor* 0.56, *A. domesticus* 0.51, *H. illucens* 0.41), indicative of relatively condensed molecular structures frequently associated with more recalcitrant organic matter pools [84]. By contrast, EFV displayed a more balanced molecular distribution of ~30% carbohydrates, ~30% proteins, 26.43% lignin, and 13.02% aliphatic, indicative of a highly processed, stabilized matrix consistent with advanced humification and microbial transformation. A distribution of ~5.6% carbonyls further supports oxidative restructuring during compost maturation. Taken together, these molecular patterns highlight the functional differences between IF and EFV.

Frass represents labile, nutrient-dense inputs suited to rapid mineralization and short-term fertilization, whereas vermicompost acts as a reservoir of chemically stabilized organic matter, which has implications for soil C sequestration and long-term fertility. This organism-linked partitioning-simpler residues from insect digestion versus chemically diversified, microbially stabilized matrices from vermicompost is central for tailoring amendment strategies (nutrient supply versus carbon stabilization).

### 4.5. Methodological Considerations and Broader Implications

The successful application of the model to these substrates reinforces its utility for estimating gross molecular composition from ^13^C NMR data [38]. By partitioning signals into biologically relevant domains (alkyl, O-alkyl, aromatic and carbonyl), the model provides a practical link between spectra and function. The close match between the predicted and observed spectral distributions reported in previous studies of fresh residues, composts, and humified materials supports the robustness of the model and justifies its use for IF-EFV comparisons in this study [30]. More broadly, our interpretation aligns with the contemporary view that soil and compost organic matter are best conceptualized as ensembles of identifiable molecular components, rather than as classical “humic substances” [51,85]. This perspective explains why species identity and process history result in coherent, mechanistically interpretable differences in IF, and why, after intensive microbial processing, EFV converges towards more humified, functionally stable signatures.

### 4.6. Evaluation of Spectral Figures

The stacked ^13^C NMR spectra (Figure 2) visually corroborate the quantitative integrals and model estimates. Offset plots clearly separate polysaccharide-rich IF from more aromatic/oxidized substrates (e.g., humus and manures), and the annotated functional regions emphasize shifts in signal density across the O-alkyl (carbohydrate), alkyl (lipid/chitin), aromatic (lignin- and melanin-like), and carbonyl domains. This visualization was instrumental in interpreting structural transformation along the labile-recalcitrant continuum. Using trapezoidal numerical integration to compute regional integrals improved comparability among spectra by minimizing bias relative to simple summation, thereby refining cross-sample contrasts in a way that is consistent with best practice in solid-state NMR quantification [50,86]. Complementing the NMR-based interpretation, FTIR features of the EFV further corroborated its advanced humification state. The EFV spectrum showed distinct bands at ~871.6 and ~1414.5 cm^−1^, which are consistent with carbonate (CO_3_^2−^) groups and aliphatic CH_2_/CH_3_ deformations, respectively. These are signals that are typical of humified organic matter and are indicative of extensive biochemical processing and structural rearrangement [87,88,89]. Additionally, the relative weakness of amide bands, particularly near ~1530 and ~1630 cm^−1^, suggests advanced degradation of proteinaceous material, a hallmark of mature composts [90].

### 4.7. Degradability and Thermal Stability (R_1_, R_2_ Continuum)

Calorimetric–thermal indices captured a stability continuum closely tied to species identity. *G. mellonella* and *A. domesticus* concentrated a larger fraction of mass/enthalpy in high-temperature domains (higher R_1_ and/or R_2_), indicating thermally recalcitrant pools and slower degradation. *T. molitor* showed a low R_1_ (~0.50), consistent with a labile matrix prone to rapid biodegradation. The EFV exhibited intermediate stability, consistent with its humified nature [69,91]. However, differences between Tmax_1_ and Tmax_2_, together with the relative exothermic areas (Area_2_/Area_1_), refine the interpretation of compound classes. In *G. mellonella*, higher Tmax and R_2_ indicate structurally complex, recalcitrant fractions, whereas in the EFV, they are more consistent with microbially humified material. These findings emphasize the complementary roles of TGA and DSC in assessing organic-matter stability. R_1_ serves as a rapid indicator of recalcitrance, while R_2_ and Tmax provide mechanistic insight into the energy requirements and compositional drivers of thermal resistance. In *T. molitor*, a high Tmax_2_ implies that some structural components resist thermal degradation; however, the predominance of heat release at lower temperatures (Area_1_ = 604.57; Area_2_ = 551.67) indicates a labile composition dominated by easily oxidizable compounds, which is consistent with its classification as a nutrient-rich but rapidly mineralizable input. Together, these thermal parameters form a diagnostic framework with which to distinguish the functional maturity and ecological roles of insect-derived residues versus composted materials. Such materials may resist microbial mineralization, resulting in slow-release behavior and longer residence times in the soil. Conversely, the low R_1_ of *T. molitor* supports its use as a fast-acting amendment in nutrient-limited systems. Positioning EFV within this continuum clarifies its intermediate R_1_. EFV contains both labile and recalcitrant fractions, reflecting biochemical heterogeneity and microbial humification. Unlike IF, which often retains native biopolymers, EFV stabilized through microbial transformation and oxidative restructuring. This ordering mirrors spectroscopic contrasts and links composition to function: *G. mellonella* is conditioner-like, *T. molitor* is fast-mineralizing, *A. domesticus* is mixed but influenced by ionic strength and *H. illucens* is partially stabilized with a notable high-temperature fraction [50,87]. For *A. domesticus*, this pattern suggests a substantial fraction of thermally recalcitrant organic matter, reinforcing its classification as a moderately stabilized substrate. However, it is important to note that high R-indices alone do not equate to agronomic “maturity”: condensed yet biologically inert domains can increase thermal recalcitrance without alleviating short-term plant stressors if EC or low-MW acids remain elevated [92,93,94]. Therefore, R_1_ is most informative when considered alongside chemical and microbial indicators. A high R_1_ does not necessarily indicate compost maturity, and a lower R_1_ (as observed in *T. molitor*) may suggest greater biodegradability despite reduced thermal resistance [95,96,97].

### 4.8. Eco- and Phytotoxicity

Bioassays translated the above chemistry into integrated biological signals. *T. molitor* and *A. domesticus* extracts exhibited strongly phytotoxic (GI < 30%), which is consistent with (i) the high labile-C pressure and ammonium/organic acid loads in *T. molitor* and (ii) the soluble salts/ionic stress in *A. domesticus*. By contrast, *H. illucens* and *G. mellonella* were non-phytotoxic under the same extract conditions, and EFV consistently exhibited the highest %GI, consistent with its maturity. As some materials intended for biofertilizer formulations were found to inhibited germination and/or root elongation, dilution assays were performed to quantify attenuation of these effects and derive practical guidelines for product design. Across diluted and undiluted extracts, %GI increased with dilution for all materials, especially for *T. molitor* and *A. domesticus*, to a lesser extent for *H. illucens* frass, and not significantly for *E. fetida*. For *T. molitor*, GI reached 50.7% at a dilution of 1:2 and 67.1% at a dilution of 1:5 (from an initial baseline of ~1%), indicating a strong dose-dependent effect. Ecotoxicity (TU) was low overall, but highest in *T. molitor* (approximately 1.47 TU), which aligns with a labile, oxygen-demanding matrix. Although TU values were not high, they should be considered when making recommendations for use; dilution or blending reduces the risk of acute toxicity. In practice, dilution improved %GI in all scenarios. Based on the phytotoxicity-limiting factor, it is advisable to include. *T. molitor* frass at a concentration of 20–30% in blends. In general, the participation of IF in complex formulations at >20% may induce phytotoxicity; however, combining it with phytostimulant materials could allow for a higher inclusion without significant risk. EFV can function as a buffering component to reduce overall phytotoxicity in frass-derived biofertilizers. Extending to seed bioassays, %GI effectively highlights risk. *T. molitor* and *A. domesticus* should be considered phytotoxic, and their inclusion rates should be optimized in future formulations. The remaining materials did not exhibit show phytotoxic effects under the tested conditions. EFV demonstrated the highest %GI, reinforcing its role as a maturity reference and blending partner. Furthermore, several studies report improved crop production and physiological status when these products are applied, emphasizing the importance of dosage and formulation when translating phytotoxicity/ecotoxicity into agricultural practice.

### 4.9. An Integrated Framework

This study reveals a three-axis framework: (1) EC (electrical conductivity) modulates osmotic/ionic stress; (2) the availability of labile-C, e.g., DOC, as proxied by C/N and low-T exotherms, governs short-term microbial O_2_ and N dynamics; and (3) structural stability (R_1_/R_2_, aromaticity) sets the tempo of carbon turnover. The results clearly differentiate IF from the EFV, highlighting distinct molecular features and potential agronomic roles. High levels of labile organic matter can restrict the agricultural use of frass, particularly that from *Tenebrio* larvae. Ref. [98] reported that applying immature compost with high labile-C content to soil induces a rapid increase in microbial activity, which can enhance the mineralization of native soil organic matter or even lead to microbial N sequestration. Fulvic and humic carbon values in insect frass were higher values than in EFV, which could increase the economic value of this material as a fertilizer. Mapping IF onto this space explains both interspecific ranking and its divergence from EFV, reconciling heterogeneous findings across prior studies by making species identity and process history explicit [56,57]. Within the broader circular bioeconomy context, this species-resolved view clarifies how IF can complement—rather than replace—composted amendments. IF provides targeted nutrient release, while EFV/humified inputs supply structural stability [73,74].

### 4.10. Future Research Directions

Two potential future lines of research could be: (i) species-specific dose–response trials under different soil conditions (e.g., texture and salinity levels) to study the relationship of EC/DOC/R (electrical conductivity/dissolved organic carbon/respiration) indices with soil characteristics or processes over time; and (ii) studies related to feedstock and post-processing to adjust the balance between rapid nutrient release and biosafety [72,73].

## 5. Conclusions

The insect frass that has been studied are not uniform products; their agronomic properties, composition, stability, and associated risks can vary significantly depending on the species. *A. domesticus* frass is rich in N, P, and K and it exhibits high electrical conductivity. *T. molitor* frass contains high levels of labile organic carbon and can be phytotoxic. Frass from *G. mellonella* and *H. illucens* were non-phytotoxic; the former was notable for its high thermal stability and the latter for its lowest ecotoxicity. Structurally, frass is minimally processed organic matter that is rich in carbohydrates and proteins, making it a fast-acting fertilizer. In contrast, vermicompost is a stable, humified and stable matrix that functions as a long-term soil conditioner. Therefore, the direct or uncontrolled application of *T. molitor* and *A. domesticus* frass is not recommended due to the risk of phytotoxicity risks. To validate new by-products from insect farms as safe and effective biofertilizers, field trials or soil-based trials need to be conducted. However, based on laboratory indicators, the most effective strategy to avoid phytotoxic responses is to formulate blends by combining this frass in controlled proportions with stabilizing materials, such as *E. fetida* vermicompost. This synergy could enable rapid nutrient release from the frass, while mitigating toxicity risks and improving long-term soil health. This would transform an industrial by-product into a safe and effective biofertilizer.

## Figures and Tables

**Table 1:** Characterization of four insect frass samples (IF) and an insect frass average sample (IFA) compared to *E. fetida* vermicompost (EFV).

Insect Frass									
Parameter	*T.* *molitor* (*n* = 6)	*G.* *mellonella* (*n* = 4)	*H.* *illucens* (*n* = 8)	*A.* *domesticus* (*n* = 12)	*E. fetida* (*n* = 10)	*F-ANOVA*	IFA (*n* = 30)	EFV (*n* = 10)	*F-ANOVA*
C (%)	39.6 d	41.9 e	37.2 c	34.8 b	25.3 a	175 ***	38.6 b	25.3 a	75 ***
N (%)	3.6 bc	4.0 c	2.9 ab	6.4 d	2.4 a	59 ***	4.1 b	2.4 a	17 **
P (%)	1.20 b	0.77 a	0.92 a	1.44 c	0.81 a	23 ***	1.10 a	0.81 a	0.5 ns
K (%)	1.54 c	0.99 a	2.05 d	3.02 e	1.28 b	38 ***	1.83 a	1.28 a	0.7 ns
Ca (%)	0.19 a	0.59 b	0.28 a	2.21 c	2.93 d	1026 ***	0.69 a	2.93 b	6.9 ***
Mg (%)	0.47 c	0.15 a	0.27 b	0.66 d	0.44 c	524 ***	0.40 a	0.44 a	0.6 ns
Na (%)	0.03 a	0.24 c	0.05 b	0.60 d	0.21 c	20,340 ***	0.19 a	0.21 a	2.2 ns
S (%)	0.21 a	0.23 b	0.27 c	0.53 d	0.31 c	1429 ***	0.29 a	0.21 a	1.9 ns
Si (%)	0.22 a	1.32 b	0.04 a	0.04 a	0.15 b	54 ***	0.37 b	0.15 a	4.1 **
B (ppm)	117 c	756 d	14 a	33 a	55 b	55 ***	208 a	55 b	2.1 ns
Fe (ppm)	232 b	42 a	437 c	871 d	6575 e	679 ***	363 a	6575 b	971 ***
Cu (ppm)	14.8 b	4.3 a	17.5 c	66.2 d	75.1 e	6232 ***	23.5 a	75.1 b	361 ***
Mn (ppm)	167 c	39 a	91 b	473 e	347 d	163 ***	187 a	347 b	96 ***
Zn (ppm)	94 b	57 a	103 b	587 d	287 c	1592 ***	187 a	287 b	225 ***
Al (ppm)	212 a	306 ab	449 b	871 c	390 b	51 ***	410 a	390 a	0.3 ns
Mo (ppm)	1.41 b	0.27 a	2.17 c	6.37 d	2.8 c	925 ***	2.3 a	2.8 a	2.1 ns
Cd (ppm)	0.12 c	0.06 b	0.04 a	0.17 d	0.40 e	117 ***	0.10 a	0.40 b	63 **
Ni (ppm)	1.2 a	1.8 b	2.6 c	9.4 d	10.3 d	3697 ***	3.2 a	10.3 b	29 **
Cr (ppm)	1.5 a	2.4 b	4.4 c	11.0 d	27.1 e	724 ***	4.2 a	27.1 b	230 ***
Pb (ppm)	0.43 ab	0.01 ab	0.95 b	1.03 b	12.2 c	45 **	0.57 a	12.2 b	567 ***
Co (ppm)	0.43 ab	0.1 ab	0.95 b	1.03 b	1.8 c	14 ***	0.15 a	1.8 b	12 **
Ti (ppm)	8.5 a	7.3 a	12.6 a	27.1 b	15 a	17 **	13 a	15 a	1.3 ns
Li (ppm)	0.1 a	1.3 a	1.5 a	1.9 a	3.1 b	9.1 **	1.2 a	3.1 b	6.6 *
Be (ppm)	0.02 a	0.16 b	0.05 a	0.05 a	0.21 b	12 **	0.06 a	0.21 c	8.3 **
Sr (ppm)	9.1 b	6.0 a	15.9 c	36.9 d	25.1 cd	212 ***	15.4 a	25.1 b	5.3 **
Rb (ppm)	9.9 a	11.1 a	10.6 a	25.4 b	31 b	339 ***	13.4 a	31 b	11 **

Different letters within a row indicate statistically significant differences between insect species and EFV (left) and average for all insect species and EFV (right) for each parameter. Significance: ns = not significant, * = *p* < 0.01, ** *p* < 0.001, *** = *p* < 0.0001.

**Table 2:** Physicochemical characteristics of IF and EFV.

IF	pH	EC (dS m^−1^)	OM (%)	C/N	DOC (g kg^−1^)	C_HA_ (%)	C_FA_ (%)
*T. molitor* (*n* = 6)	5.66 a	5.32 b	88.9 c	11.3 bc	74.1 d	8.10 d	9.05 d
*G. mellonella* (*n* = 4)	6.74 b	4.81 a	82.0 b	10.4 b	36.1 b	8.60 e	9.60 e
*H. illucens* (*n* = 8)	7.55 c	4.74 a	90.1 c	12.6 c	49.9 c	7.63 c	8.53 c
*A. domesticus* (*n* = 12)	6.49 b	9.59 c	86.4 b	5.4 a	35.3 b	7.13 b	7.96 b
EFV (*n* = 10)	7.48 c	5.42 b	52.9 a	10.2 b	8.84	3.98 a	2.93 a
*F-ANOVA*	*113* ***	*238* ***	*259* ***	*23* ***	*1097* ***	*2718* ***	*2230* ***
IF Average (*n* = 30)	6.53 a	6.05	87.0	10.2 a	50.8 b	7.88 b	8.81 b
EFV (*n* = 10)	7.48 b	5.42	52.9	10.2 a	8.83 a	3.9 a	2.93 a
*F-ANOVA*	*8.52* **	*0.5* ns	*438* ***	*0.2* ns	*34* ***	*287* ***	*399* ***

EC: electrical conductivity, OM: organic matter, C/N: carbon/nitrogen ratio, DOC: dissolved organic carbon, C_FA_: carbon in fulvic acids, C_HA_: carbon in humic acids. Different letters within a column indicate statistically significant differences between insect species and EFV (top) or between the mean IF and EFV (bottom) for each parameter. Significance: ns = not significant, ** = *p* < 0.001, *** = *p* < 0.0001.

**Table 3:** Estimated biomolecular composition of samples based on model by [38]. Values represent percentage of organic carbon attributed to carbohydrates, proteins, lignin, aliphatic compounds, and carbonyl structures. These estimations are derived from mathematical fitting of nuclear magnetic resonance (NMR) spectral distributions to spectra of model components.

IF	Carbohydrates	Protein	Lignin	Aliphatic	Carbonyl
*T. molitor*	64.23	21.73	12.98	2.27	0.00
*G. mellonella*	58.92	26.40	8.93	0.00	5.06
*H. illucens*	66.01	24.87	4.75	3.24	0.51
*A. domesticus*	62.38	29.29	7.07	0.11	1.48
EFV	29.71	27.83	26.43	13.02	5.58

**Table 4:** Phytotoxicity indexes: percentage of germination (%G), percentage of radicular length (%L), and germination index (%GI) of insect frass and *E. fetida* vermicompost (EFV).

IF	%G	%L	%GI
*T. molitor*	13.8 a	8.8 a	1.3 a
*G. mellonella*	99.1 c	73.9 c	72.6 c
*H. illucens*	85.4 bc	81.2 c	69.7 c
*A. domesticus*	70.3 b	40.7 b	28.6 b
EFV	100 c	128 d	129 d
*F-ANOVA*	63 ***	229 ***	140 ***

Different letters within a column indicate statistically significant differences between insect species and EFV for each parameter. Significance: *** = *p* < 0.0001.

## Data Availability

The original contributions presented in this study are included in the article/Supplementary Materials. Further inquiries can be directed to the corresponding author.

## Supplementary Materials

The following supporting information can be downloaded at: https://www.mdpi.com/article/10.3390/insects17020142/s1, Table S1. Normalized integrals of ^13^C CP-MAS NMR spectral regions for insect frass (IF) and *E. fetida* vermicompost (EFV) samples. Table S2. Mass loss percentages and R_1_ values of insect frass (IF) and *E. fetida* vermicompost (EFV) samples, as derived from thermogravimetric analysis. Mass loss was calculated for temperature intervals of 200–400 °C and 400–550 °C, which correspond to the decomposition of distinct organic fractions. Table S3. Differential scanning calorimetry (DSC) peak temperatures, exothermic areas and R_2_ values for insect frass (IF) and *E. fetida* vermicompost (*EFV*) samples. Figure S1. Estimated biomolecular composition of organic carbon in each sample, derived from solid-state ^13^C CP-MAS NMR spectra using the mixing model [30]. The stacked bar chart illustrates the proportionate contributions of carbohydrates, proteins, lignin, aliphatic compounds, and carbonyl structures to the total organic carbon content. Variations between samples reflect differences in biochemical origin, degree of decomposition, and organic matter transformation pathways.

## Abbreviations

The following abbreviations are used in this manuscript:

IF	Insect frass
EFV	*Eisenia fetida* vermicompost
GI	Germination index
EC_50_	Effective concentration 50%
TU	Toxicity units
NMR	Nuclear magnetic resonance
CP-MAS	Cross-polarization magic angle spinning
FTIR	Fourier-transform infrared spectroscopy
ATR	Attenuated total reflection
TG	Thermogravimetry
DTG	Derivative thermogravimetry
TGA	Thermogravimetric analysis
DSC	Differential scanning calorimetry
Tmax	Temperature of maximum signal
Tmax_1_	Temperature of first maximum signal
Tmax_2_	Temperature of second maximum signal
R_1_	Thermal stability ratio (mass loss 400–550 °C/200–400 °C)
R_2_	Calorimetric ratio (DSC Area_2_/Area_1_)
EC	Electrical conductivity
OM	Organic matter
DOC	Dissolved organic carbon
C_HA_	Carbon in humic acids
C_FA_	Carbon in fulvic acids
TOM	Total organic matter
TN	Total nitrogen
TOC	Total organic carbon
NaOH	Sodium hydroxide
ICP-OES	Inductively coupled plasma–optical emission spectrometry
%G	Germination percentage
%L	Root length percentage
%GI	Germination index percentage
