# Supplementary material for: Could Insect Frass Be Used as a New Organic Fertilizer in Agriculture? Nutritional Composition, Nature of Organic Matter, Ecotoxicity, and Phytotoxicity of Insect Excrement Compared to Eisenia fetida Vermicompost
Source: Insects. 2026 Jan 27;17(2):142. doi: 10.3390/insects17020142 (PMC12941309; doi:10.3390/insects17020142)
Supplement: Supplementary file 1 [file insects-17-00142-s001.zip › insects-4098791-supplementary.pdf]

Supplementary Materials

Table S1. Normalized integrals of ^{13}C CP-MAS NMR spectral regions for insect frass (IF) and *E. fetida* vermicompost (EFV) samples.

IF	Alky l C	O/N Alkyl C	Methoxy l C	Anome ric C	Aromat ic C	Phenol ic C	Carboxyl ic C	Carbonyl C	Arom ati- city Index
T. molitor	11.70	4.03	53.09	16.02	5.41	3.35	3.83	2.58	0.56
G. mellonella	11.41	3.14	48.29	13.12	5.80	5.80	6.73	5.72	0.80
H. illucens	12.82	4.08	53.60	15.07	4.54	2.34	4.47	3.07	0.41
A. domesticus	12.44	4.06	50.84	14.65	4.72	3.77	5.11	4.41	0.51
EFV	23.70	9.92	28.55	8.59	9.03	5.35	8.67	6.19	0.43

The values represent the proportion of the total ^{13}C signal intensity (%) attributed to each chemical shift domain, including alkyl (0–45 ppm), O/N-alkyl (45–110 ppm), aromatic (110–160 ppm), and carbonyl (160–220 ppm). The aromaticity index is calculated as the ratio of aromatic to aliphatic (i.e. alkyl and O/N-alkyl) carbon.

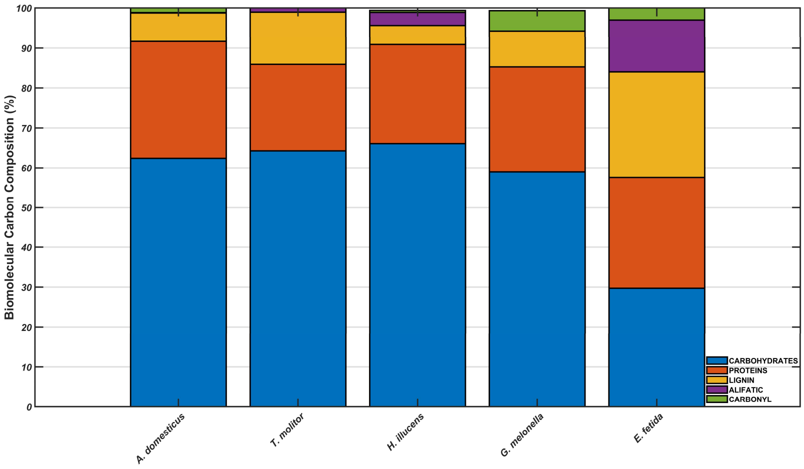

Figure S1. Estimated biomolecular composition of organic carbon in each sample, derived from solid-state ^{13}C CP-MAS NMR spectra using the mixing model [30]. The stacked bar chart illustrates the proportionate contributions of carbohydrates, proteins, lignin, aliphatic compounds, and carbonyl structures to the total organic carbon content. Variations between samples reflect differences in biochemical origin, degree of decomposition, and organic matter transformation pathways.

Table S2. Mass loss percentages and R_1 values of insect frass (IF) and *E. fetida* vermicompost (EFV) samples, as derived from thermogravimetric analysis. Mass loss was calculated for temperature intervals of 200- 400 °C and 400- 550 °C, which correspond to the decomposition of distinct organic fractions.

IF	Loss 200- 400 ºC	Loss 400- 550 ºC	R_1
T. molitor	45.62	22.89	0.50
G. mellonella	36.91	32.36	0.88
H. illucens	41.73	30.34	0.73
A. domesticus	34.71	27.29	0.79
EFV	20.47	13.79	0.67

R_1 is calculated by dividing the mass loss in the 400–550 °C by the mass loss in the 200–400 °C range. It provides an indication of the thermal stability and recalcitrance of the samples in question.

Table S3. Differential scanning calorimetry (DSC) peak temperatures, exothermic areas and R_2 values for insect frass (IF*) and E. fetida* vermicompost *(EFV)* samples.

IF	$Tmax_1$	$Tmax_2$	$Area_1$	$Area_2$	R_2
T. molitor	297.00	523.67	604.57	551.67	0.91
G. mellonella	327.67	549.00	128.78	327.96	2.55
H. illucens	282.33	425.00	398.17	985.24	2.47
A. domesticus	302.33	495.67	328.79	781.22	2.38
EFV	331.67	490.33	371.44	350.09	0.94

$Tmax_1$ and $Tmax_2$ represent the temperatures (°C) at which the maximum heat flow was observed during the decomposition of the labile (200–400 °C) and recalcitrant (400–550 °C) organic fractions, respectively. $Area_1$ and $Area_2$ correspond to the integrated heat release (mW·s) in each thermal range. The R_2 index is calculated as the ratio of $Area_2$ to $Area_1$, reflecting the relative dominance of high-temperature exothermic reactions, associated with structurally resistant organic matter.
